# Modeling adaptation of sorghum in Ethiopia with APSIM—opportunities with G×E×M

**DOI:** 10.1007/s13593-023-00869-w

**Published:** 2023-01-24

**Authors:** Alemu Tirfessa, Fikadu Getachew, Greg McLean, Erik van Oosterom, David Jordan, Graeme Hammer

**Affiliations:** 1 Ethiopian Institute of Agricultural Research (EIAR), Melkassa Agricultural Research Center, P.O. Box 436, Adama, Ethiopia; 2 Institute of Food and Agricultural Sciences, University of Florida, Gainesville, FL USA; 3 Queensland Alliance for Agriculture and Food Innovation (QAAFI), The University of Queensland, St Lucia, QLD Australia; 4 Queensland Alliance for Agriculture and Food Innovation (QAAFI), Hermitage Research Facility, The University of Queensland, Warwick, QLD Australia

**Keywords:** Sorghum, Model, Simulation, Crop adaptation, Land race, Ethiopia, G×E×M

## Abstract

Sorghum is an important food and feed crop in the dry lowland areas of Ethiopia. Farmers grow both early-sown long-duration landraces and late-sown short-duration improved varieties. Because timing and intensity of drought stress can vary in space and time, an understanding of major traits (G), environments (E), management (M), and their interactions (G×E×M) is needed to optimize grain and forage yield given the limited available resources. Crop simulation modeling can provide insights into these complex G×E×M interactions and be used to identify possible avenues for adaptation to prevalent drought patterns in Ethiopia. In a previous study predictive phenology models were developed for a range of Ethiopian germplasm. In this study, the aims were to (1) further parameterize and validate the APSIM-sorghum model for crop growth and yield of Ethiopian germplasm, and (2) quantify by simulation the productivity-risk trade-offs associated with early vs late sowing strategies in the dry lowlands of Ethiopia. Field experiments involving Ethiopian germplasm with contrasting phenology and height were conducted under well-watered (Melkassa) and water-limited (Miesso) conditions and crop development, growth and yield measured. Soil characterization and weather records at the experimental sites, combined with model parameterization, enabled testing of the APSIM-sorghum model, which showed good correspondence between simulated and observed data. The simulated productivity for the Ethiopian dry lowlands environments showed trade-offs between biomass and grain yield for early and late sowing strategies. The late sowing strategy tended to produce less biomass except in poor seasons, whereas it tended to produce greater grain yield except in very good seasons. This study exemplified the systems approach to identifying traits and management options needed to quantify the production-risk trade-offs associated with crop adaptation in the Ethiopian dry lowlands and further exemplifies the general robustness of the sorghum model in APSIM for this task.

## Introduction

**Fig. 1:**
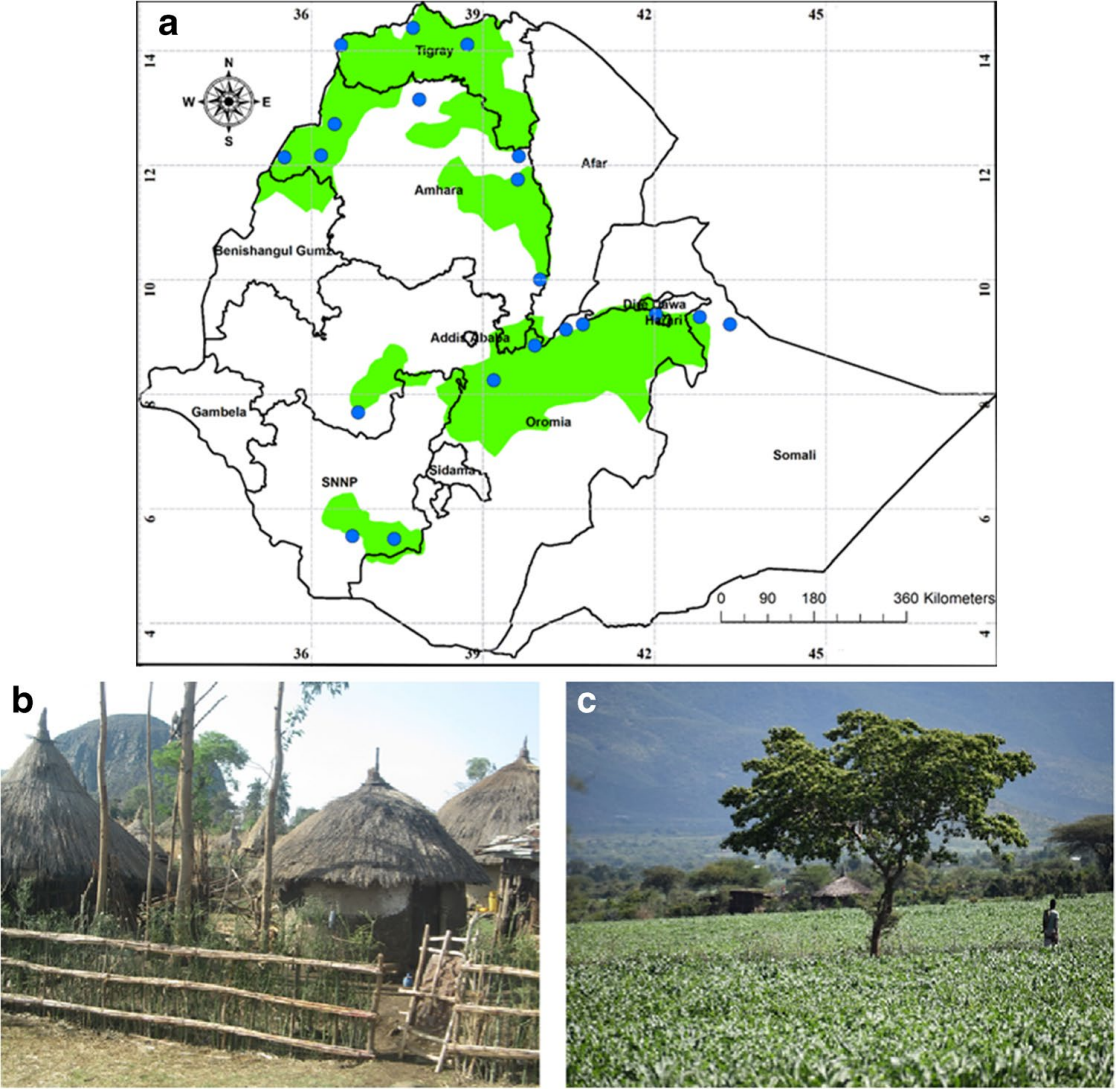
**a** Map showing dry lowland sorghum growing areas (shaded green) and the major administrative regions in Ethiopia. Most dry lowland sorghum is grown in the four regions—Tigray, Amhara, Oromia, and Southern (SNNP). Locations of key weather stations are indicated by blue dots. **b** Traditional farmhouse in the cropping region. **c** Sorghum field near Miesso, Oromia.

Sorghum remains an important food security crop in Ethiopia, covering an area of 1.8 million hectares of land, with production of 4.7 million tons of grain (CSA 2017). In the dry lowland sorghum growing areas of Ethiopia (Fig. 1), sorghum production is mainly rainfed and farmers depend entirely on sorghum for both grain and stover in these crop-livestock mixed farming systems. Sorghum grain is mostly produced for household consumption and only a small portion of production is marketed and sold. In the rural areas of Ethiopia, the stover is used for diverse purposes including fuel for cooking and heating, animal feed, and construction materials.

**Fig. 2:**
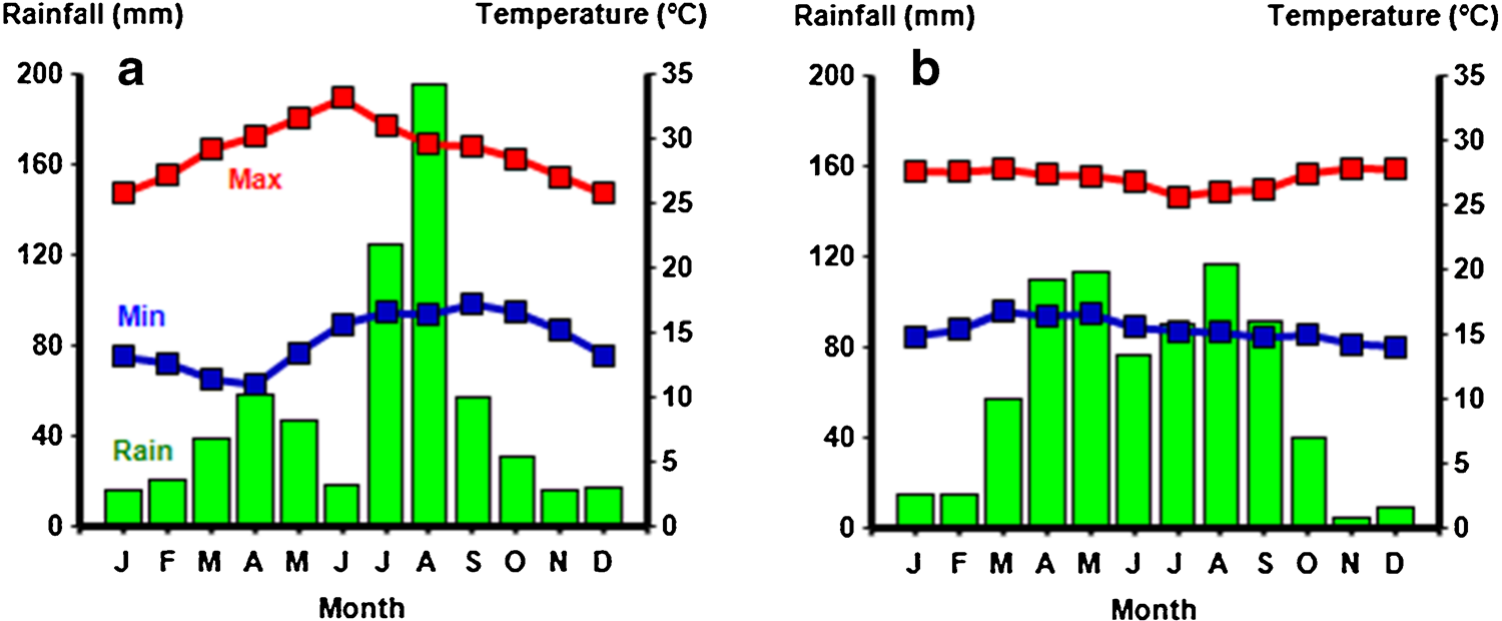
Average monthly rainfall (filled bars) and maximum and minimum temperatures (red and blue symbols) in representative dry lowland sorghum growing areas of Ethiopia: **a** Kobo in Amhara region, and **b** Babile in Oromia Region.

Although the multipurpose sorghum landraces play a significant role in the crop-livestock mixed farming system, the crop faces considerable challenges, particularly from drought stress associated with delay in on-set of rains, dry spells after sowing, and drought stress during critical crop stages. Hence, sorghum production is at risk in many instances and crop failure is a common phenomenon. In some parts of the dry lowlands, there are two distinct planting opportunities depending on the timing of rain (Fig. 2). Most farmers traditionally plant late-maturing landraces in April after 3–4 rain showers, depending on the on-set of the rain, and harvest in November. However, after planting on early rains, dry spells may occur in May and June and the landraces are often exposed to water limitation. If the dry spell is severe, the crop will completely fail. In such circumstances, depending on the availability of improved seeds of early-maturing varieties, farmers will replant with early-maturing genotypes in July and harvest in November. While there is also the option to forgo the risky early sowing and just plant early-maturing genotypes later in the season, this is rarely practiced.

The use of crop modeling as a tool to support crop management decisions has been discussed in many previous studies (Hammer et al. 2002; Stephens and Middleton 2002; Meinke et al. 2001). Furthermore, Hammer et al. (2019) and Whish et al. (2005) have also discussed the use of crop modeling as a technology for assessing production-risk trade-offs for management and adaptive trait options in the water-limited sorghum growing areas of NE Australia. In a situation like the dry lowland areas of Ethiopia, crop simulation modeling has the potential to play a critical support role for assessing production-risk trade-offs confronted by sorghum farmers with sowing decision options. Hence, in this study, the objectives are to (i) parameterize and validate the APSIM-sorghum model for the prediction of growth and yield of Ethiopian germplasm, and (ii) use the model in simulation studies to quantify productivity-risk trade-offs associated with early planting of late-maturing landraces and late planting of early-maturing lines in the dry lowland sorghum growing areas of Ethiopia. The generality of the biological functionality underpinning the APSIM-sorghum model (Hammer et al. 2019; Holzworth et al. 2014) is discussed in relation to how it facilitates studies of this nature.

## Materials and methods

### Experiments to characterize growth and yield of Ethiopian germplasm

Developmental responses of key Ethiopian sorghum germplasm have been quantified for modeling (Tirfessa et al. 2020), however, no information is available for parameterizing and testing models of growth and yield for that germplasm. To generate this information, growth analysis experiments were conducted at Melkassa under non-limiting (water and nitrogen) conditions in 2014 and at Miesso for water-limited dryland conditions in 2016. Data from analysis of previous phenology experiments (Tirfessa et al. 2020) were used to derive canopy development coefficients (Tirfessa et al. 2022), whereas crop growth coefficients were derived from the growth analysis experiments of this study. The genotypes used in the growth analysis experiments represented a subset of those used in the phenology experiments (see Table 1).

**Table 1:** Phenology, canopy development and crop growth parameters used in the APSIM-sorghum crop model for the two reference genotypes, Meko and Jigurti. After anthesis the rate of development reaches a plateau at *T*_opt_ and does not decline at higher temperatures as is the case for development prior to anthesis (Hammer and Muchow 1994).

Category	Parameter	Meko	Jigurti
Phenology**—**emergence to anthesis	*T*_base_ (°C)	6.0	6.6
	*T*_opt_ (°C)	27	27
	*T*_max_ (°C)	42	42
Phenology**—**emergence **t**ofloral initiation	Accumulated thermal time target (°Cd)	347	450
Phenology—flag leaf full expansion to anthesis	Accumulated thermal time target (°Cd)	207	222
Phenology**—**anthesis to physiological maturity	*T*_base_ (°C)	5.7	5.7
	*T*_opt_ (°C)	23.5	23.5
	Accumulated thermal time target (°Cd)	819	801
Canopy development	Leaf appearance rate (LAR; °Cd leaf^−1^) Leaf initiation rate (LIR; °Cd leaf^−1^)	63 31.5	58 29
Crop growth	Leaf-stem partitioning factor Grain number factor (g grain^−1^) Radiation Use Efficiency (RUE; g MJ^−1^)	0.0073 0.00083 1.25	0.0106 0.00140 1.65

#### Experimental details

Five genotypes representing landraces (ESH2, Gambella1107, Jigurti), an improved hybrid (Teshale), and an improved variety (Meko), were planted in a randomized complete block design with three replications under non-limiting (water and nitrogen) conditions at Melkassa on 9 June 2014. The recommended rate of phosphorus fertilizer (46 kg/ha P_2_O_5_) in the form of Di-Ammonium Phosphate (DAP) and nitrogen fertilizer (23 kg/ha nitrogen in the form of urea) was applied at planting and at 35 days after planting. The experimental plots were irrigated every 5 days using furrow irrigation to ensure water was not limiting. Additional nitrogen fertilizer (at rate of 23 kg/ha nitrogen in the form of urea) was applied at 50 days after planting in order to ensure nitrogen was not limiting. The same genotypes were planted with four replications in Miesso on 8 July 2016 under dry land conditions. The recommended rate of phosphorus fertilizer (46 kg/ha P2O5) in the form of Di-Ammonium Phosphate (DAP) and nitrogen fertilizer (23 kg/ha nitrogen in the form of urea) was applied at planting and at 35 days after planting respectively for the experiment at Miesso. For both experiments, each genotype was planted in a plot of 10 rows of 5 m length with 0.75 m row spacing and 0.15 m between plants, giving a planting density of 8.9 plants per m^2^. Seeds were manually drilled into the rows and at about 20 days after emergence, plants were thinned to 0.15 m distance between plants.

#### Biomass sampling

Aboveground biomass was determined on four occasions by destructively sampling a quadrat of 1 m^2^ in each plot at the stages of eight leaves fully expanded (i.e., ligule of leaf 8 visible), flag leaf fully expanded, anthesis, and physiological maturity. Due to unforeseen problems (i.e., local political instability), no biomass sample could be taken for the late-maturing genotype (Jigurti) at maturity in Miesso. Plants were cut at ground level and fresh weight for all the plants was measured. The fresh weight for a representative subsample of five plants was then taken. The five plant subsample was separated into the stem (including sheaths), green leaves, dead leaves, and panicles. The dry weight for each component was obtained after drying samples at 70 °C for at least 3 days. Green leaf area was measured by passing all green leaves from the subsampled plants through a leaf area meter (LICOR 3100, Lincoln, NE, USA).

#### Soil characterization

Plant available water content (PAWC) of the soil was determined from field measurements of drained upper limit (DUL), crop lower limit (CLL), and bulk density (BD) for the soils at Melkassa and Miesso experimental sites using the protocol of Burk and Dalgliesh (2008). To determine DUL and BD, a bunded 4m × 4m area adjacent to each experimental field was irrigated regularly for 4 weeks, covered with black plastic sheet, and allowed to drain for 1 to 2 weeks before coring to a depth of 1.6 m at Melkassa and 1.8 m at Miesso and extracting samples for each 15–30 cm depth increment. The wet weight of the soil sample was recorded, and the sample dried at 105 °C until a constant weight was attained. Using the sample volume (from tube diameter and length) and wet and dry weights, BD and DUL were calculated for each soil layer (Dalgliesh and Foale 1998) as:

$$\mathrm G\mathrm r\mathrm a\mathrm v\mathrm i\mathrm m\mathrm e\mathrm t\mathrm r\mathrm i\mathrm c\;\mathrm w\mathrm a\mathrm t\mathrm e\mathrm r\;\%\;\mathrm a\mathrm t\;\mathrm D\mathrm U\mathrm L=\left(\left(\mathrm{wet}\;\mathrm{wt}\;\mathrm{of}\;\mathrm{sample}-\mathrm{dry}\;\mathrm{wt}\;\mathrm{of}\;\mathrm{sample}\right)/\mathrm{dry}\;\mathrm{wt}\;\mathrm{of}\;\mathrm{sample}\right)\times100$$

$$\mathrm{BD}\;\left(\mathrm{gcm}^{-3}\right)=\mathrm{dry}\;\mathrm{soil}\;\mathrm{wt}\;\left(\mathrm g\right)/\mathrm{total}\;\mathrm{volume}\;\mathrm{of}\;\mathrm{soil}\;\left(\mathrm{cm}^3\right)$$

$$\mathrm{DUL}\;\left(\mathrm v\mathrm o\mathrm l\mathrm u\mathrm m\mathrm e\mathrm t\mathrm r\mathrm i\mathrm c\;\mathrm w\mathrm a\mathrm t\mathrm e\mathrm r\;\%\right)=\mathrm G\mathrm r\mathrm a\mathrm v\mathrm i\mathrm m\mathrm e\mathrm t\mathrm r\mathrm i\mathrm c\;\mathrm w\mathrm a\mathrm t\mathrm e\mathrm r\;\%\;\mathrm a\mathrm t\;\mathrm D\mathrm U\mathrm L\times\mathrm{BD}$$

To determine CLL at Miesso, we used an experiment that was severely affected by terminal stress around flowering, to the extent the plants died. We assumed that those plants extracted all the water available in the soil, such that soil samples would provide a good estimate of CLL. At Melkassa, a site close to the enclosure used for determining DUL was identified and established for the measurement of CLL. Sorghum plants of the check variety Meko were sown in a 4m × 4m bunded area and irrigated throughout the first weeks of growth so that the soil was fully wet to depth. A transparent rain exclusion tent was then erected over the plot to allow the plants to fully dry the soil profile. Soil samples were taken at maturity of the crop to estimate CLL throughout the profile. Samples wet weights were recorded before being dried at 105 °C until a constant weight was reached and the gravimetric water % at CLL was calculated from sample weights as above for DUL. Using the BD determined from the DUL sampling, the volumetric water % at CLL was calculated as:

$$\mathrm{CLL}\;\left(\mathrm{volumetric}\;\mathrm{water}\;\%\right)=\mathrm G\mathrm r\mathrm a\mathrm v\mathrm i\mathrm m\mathrm e\mathrm t\mathrm r\mathrm i\mathrm c\;\mathrm w\mathrm a\mathrm t\mathrm e\mathrm r\;\%\;\mathrm a\mathrm t\;\mathrm C\mathrm L\mathrm L\times\mathrm{BD}$$

### Canopy development characterization

The growth experiment at Melkessa was used to derive the leaf size—leaf number relationships needed for estimating potential plant leaf area development in the crop model. Fully expanded area of each leaf of each culm was estimated by measurements of leaf blade length and maximal width on four tagged plants in each plot of the experiment (i.e., 12 plants per genotype). Blade length was measured from the ligule to the tip and blade width was measured at its maximal point. Blade area was then calculated as the product of leaf length, leaf width, and a correction factor (0.635 for flag leaves and 0.71 for all other leaves) (van Oosterom et al. 2011).

A curvilinear, bell-shaped curve was fitted to the leaf size versus leaf number data based on functions used previously for sorghum (Muchow and Carberry 1990) and maize (Muchow and Carberry 1989; Birch et al. 1998).

$$Y=Y_0\;\exp\left(a\left(X-X_0\right)^2+b\left(X-X_0\right)^3\right)$$

where *Y* is the fully expanded leaf area of individual leaves, $X\;\mathrm{is}\;\mathrm{the}\;\mathrm{leaf}\;\mathrm{number},$  *Y*_0_ is the fully expanded area of the largest leaf, *X*_0_ is the leaf number of the largest leaf, and *a* and $b$ are empirical coefficients that control respectively the breadth and skewness of the leaf area profile. In the absence of extensive data in this study, estimates of the coefficients *a* and *b* reported in the comprehensive study of Birch et al. (1998) (equations 18 and 19) were employed. As in the previous studies, estimates of *Y*_0_ and *X*_0_ were derived from linear regression on total leaf number (TLN).

### Model parameterization and validation

Model parameterization and validation were focused on two widely grown check genotypes—Jigurti, a late-maturing landrace commonly used for early sowing, and Meko, an early-maturing improved variety used for late sowing. A focus on these two genotypes was relevant to underpin the subsequent simulation comparison of early sowing using a late-maturing type with late sowing using an early-maturing type for all regions in the Ethiopian dry lowland sorghum growing areas. Parameters of the phenology prediction models for these genotypes (Table 1) were reported by Tirfessa et al. (2020). *T*_base_ represents the base temperature below which development ceases, *T*_opt_ is the optimum temperature for development, and *T*_max_ is the temperature above which development ceases. The fitted accumulated thermal time targets use these cardinal temperatures and daily temperature values between the two stages under consideration. There were no significant photoperiod effects on the rate of development. The phenology models were developed only for emergence to flowering time as the timing of panicle initiation was not determined. The thermal time target from emergence to panicle initiation was estimated from the predicted thermal time to flag leaf for the genotype, its leaf appearance rate, and anticipated total leaf number. Given that four leaf initials are present in the seed, and that leaf initiation rate (LIR) is approximately half the leaf appearance rate (LAR) (Ravi Kumar et al. 2009), it was possible to derive accumulated thermal time estimates from emergence to panicle initiation for Meko and Jigurti as 347 and 450 °Cd, respectively. Parameters for leaf appearance rate for these genotypes were set at values reported in the analysis of the phenology experiments (Tirfessa et al. 2022).

The growth analysis experiments conducted at Melkassa in 2014 with non-limiting (water and nitrogen) conditions were used to test these estimates of the crop growth parameters used in the APSIM model. The sorghum model used was modified from the released version by including a leaf canopy routine that estimates individual leaf size based on equations developed for maize by Birch et al. (1998) and uses that equation to determine crop leaf area from the estimated number of fully expanded leaves using the approach of Carberry et al. (1993) (APSIM version 7.10 r4171). Given the data available on leaf appearance and individual leaf size from associated phenology experiments (Tirfessa et al. 2022), and the paucity of time series data on crop leaf area index, this approach was needed to enable the prediction of canopy leaf area development. In the first instance, parameter values for leaf-stem partitioning, grain number determination, and radiation use efficiency (RUE) found in previous studies (Hammer et al. 2010) for short and tall sorghum genotypes were used for Meko (short) and Jigurti (tall), based on their similar height and grain size characteristics (Table 1). The growth analysis experiments at Melkassa (8°24′ N 39°19′ E) and Miesso (9°13′ N 40°45′ E) were simulated using these parameter values as input to APSIM. Available soil water was determined by taking three soil cores across the experimental field at the time of sowing and determining gravimetric water content for soil profile layers. Daily weather data was recorded at adjacent meteorological stations. Given the soil characterization, estimated growth and phenology parameters, initial soil water, and seasonal weather data, each experiment was simulated and the predicted leaf area, biomass, and yield, were compared graphically to observed experimental data throughout the crop season.

### Simulation study

Long-term simulations were conducted using available soil and weather data for both early sowing with a late-maturing type and late sowing with an early-maturing type for all regions in the Ethiopian dry lowland sorghum growing areas. For early sowing, the standard late-maturing landrace was planted in April after initial rains had occurred, which resulted in harvesting in November. For late sowing, the standard early-maturing variety was planted in early July after a planting rain, which resulted in harvesting in October. Long-term meteorological data were obtained from the National Metrological Agency of Ethiopia and NASA-Power (NASA 2015) for selected stations in the growing regions (Table 2). Long-term average meteorological data is presented for two characteristic sites in Fig. 2. Detailed soil data were obtained from the FAO soils portal and world soil information (FAO 2018; Leenaars et al. 2014) for each site (Table 2) except Melkassa, and Miesso, where soil characterization for DUL, BD, and CLL was conducted as part of this study.

**Table 2:** Latitude (Lat), longitude (Long), number of years of daily weather data, soil group (FAO 2018; Ali et al 2022), soil depth (cm), and plant available soil water capacity (PAWC mm) for locations used in the long-term simulation study. SNNPR is Southern Nations Nationalities and Peoples’ Region.

Region	Station name	Lat	Long	No. of years	Soil Group	Soil Depth (cm)	PAWC (mm)
Tigray	Humera	14°01′N	36^o^52′E	16	Vertisol	100	120
	Axum	14°11′N	38°73′E	19	Leptosol	100	133
	Shiraro	14°04′N	37°78′E	7	Vertisol	100	120
Amahara	Debarik	13°15′N	37°89′E	30	Vertisol	100	106
	Shoa Robit	10°00′N	40°00′E	5	Leptosol	100	144
	Kobo	12°16′N	39o63′E	30	Leptosol	100	113
	Sirinka	11°75′N	39°61′E	21	Leptosol	100	144
Somali	Jijiga	9°35′N	42°79′E	33	Vertisol	100	132
Oromia	Babile	9°22′N	43^o^32′E	41	Vertisol	100	132
	Miesso	9^o^13′N	40°45′E	30	Vertisol	180	365
	Metehara	12°72′N	36°41′E	19	Leptosol	100	148
	Melkassa	8°24′N	39°19′E	30	Vertisol	180	272
SNNPR	Konso	9°22′N	40°75′E	27	Leptosol	100	153
	Gato	5°47′N	37°45′E	30	Leptosol	100	153
	Kayafer	5°52′N	36°72′E	20	Leptosol	100	153

## Results and discussion

### Soil characterization

Soils at Melkassa and Miesso were characterized for plant available water capacity (PAWC) through field measurement of drained upper limit (DUL), crop lower limit (CLL), and bulk density (BD) (Table 3). In general, the heavy clay soil at Miesso had higher water-holding capacity than the silty clay loam at Melkassa. The potential plant available water in the top two layers in Miesso (46 and 45 mm) was nearly double that of Melkassa (28 and 21 mm). The total plant available water capacity for the depth of the profile was 504 mm at Miesso and 244 mm at Melkassa. Measured soil moisture at the times of sowing amounted to 185 and 232 mm of available water for Miesso and Melkessa experiments respectively.

**Table 3:** Bulk density, volumetric drained upper limit (DUL), volumetric crop lower limit (CLL), and volumetric soil water at sowing (ASW) for soil profiles at experimental sites in (a) Melkassa and (b) Miesso.

a Melkassa				
Depth (cm)	Bulk density (g/cm^3^)	DUL(mm/mm)	CLL (mm/mm)	ASW (mm/mm)
0–15	1.22	0.27	0.08	0.23
15–30	1.20	0.26	0.12	0.23
30–45	1.07	0.25	0.11	0.24
45–60	1.03	0.24	0.13	0.24
60–75	1.10	0.28	0.11	0.27
75–90	1.13	0.30	0.13	0.30
90–105	0.99	0.26	0.14	0.26
105–120	1.01	0.27	0.17	0.27
120–140	1.11	0.31	0.20	0.31
140–160	1.03	0.28	0.15	0.28
b Miesso				
Depth (cm)	Bulk density (g/cm^3^)	DUL (mm/mm)	CLL (mm/mm)	ASW (mm/mm)
0–15	1.13	0.39	0.08	0.36
15–30	1.11	0.48	0.17	0.35
30–60	1.21	0.50	0.22	0.33
60–90	1.22	0.53	0.24	0.31
90–120	1.24	0.48	0.26	0.32
120–150	1.25	0.51	0.23	0.31
150–180	1.21	0.52	0.22	0.29

### Canopy development

**Fig. 3:**
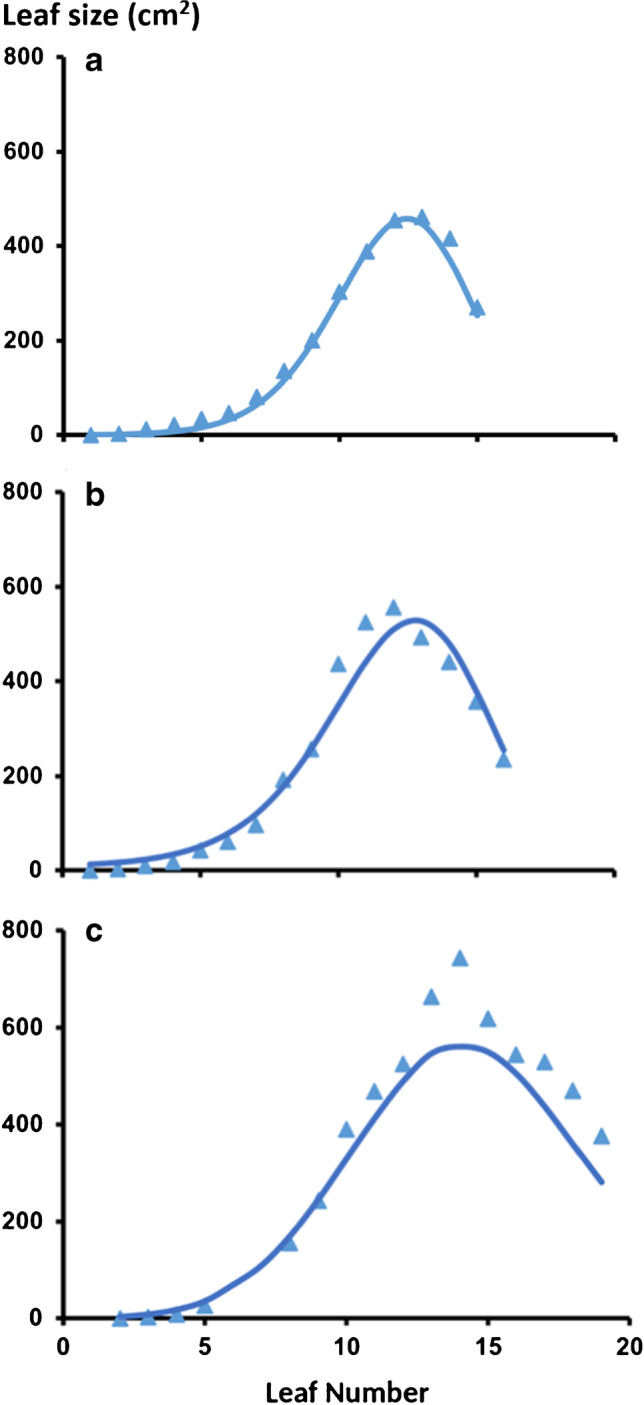
Individual leaf area versus leaf number for **a** Meko (with a total leaf number of 15) and **b**, **c** Jigurti (with a total leaf number of 16 or 19).

The curvilinear functions fitted for each genotype to quantify the leaf size distribution for plants with a given TLN fitted the observed data well for both Meko and Jigurti, although there was an underestimation around the largest leaf for the 19-leaf Jigurti (Fig. 3). Area of individual leaves increased with leaf number up to the leaf with the maximum area, which was leaf 13 for Meko (15 leaf plant) and leaf 14 (19 leaf plant) for the later-maturing Jigurti. An example for a 16-leaf plant of Jigurti is also included in Fig. 3. The linear regressions fitted on average TLN for the coefficients $X$_0_ (leaf number of largest leaf) and $Y$_0_ (area of largest leaf) were $X$_0_ = 0.83TLN (*n* = 4; R^2^ = 0.99) for Meko, $X$_0_ = 0.74TLN (*n* = 4; R^2^ = 0.99) for Jigurti, $Y$_0_ = 36.9TLN – 95.2 (*n* = 4; R^2^ = 0.99) for Meko, and $Y$_0_ = 13.2TLN + 318 (*n* = 4; R^2^ = 0.05) for Jigurti. There was a limited range in TLN among the plants sampled (4–5 leaves) and greater variability in leaf size for Jigurti, which influenced the adequacy of fit. However, the robustness of these leaf size distribution functions was consistent with findings in previous studies on sorghum (Muchow and Carberry 1990) and maize (Muchow and Carberry 1989; Birch et al. 1998). The fitted relationships between the area of individual leaves and leaf number were used to parametrize the APSIM model for predicting canopy leaf area development for Meko and Jigurti.

### Model parameterization and validation

The growth analysis experiment at Melkassa, which was conducted under non-limiting water and nutrient conditions, was used initially to test estimate model coefficients, before examining the data for the dryland experiment at Miesso, which included the effects of water limitation. In the absence of measured values, the grain number coefficient used in the model, which relates grain number to biomass accumulated during the period between panicle initiation and the start of grain filling (Rosenthal et al. 1989; Heiniger et al. 1997), was set to values previously reported (Hammer et al. 2010) for a short Australian hybrid (0.00083 g/grain) and a tall Indian landrace (0.0014 g/grain), for Meko and Jigurti respectively (Table 1). These values reflected the lower grain set per unit of crop growth by the taller Jigurti, an association also observed by van Oosterom and Hammer (2008). Values of coefficients for the stem-leaf partitioning function and RUE (Table 1) were similarly estimated from values reported for short and tall genotypes in the detailed study of Hammer et al. (2010).

**Fig. 4:**
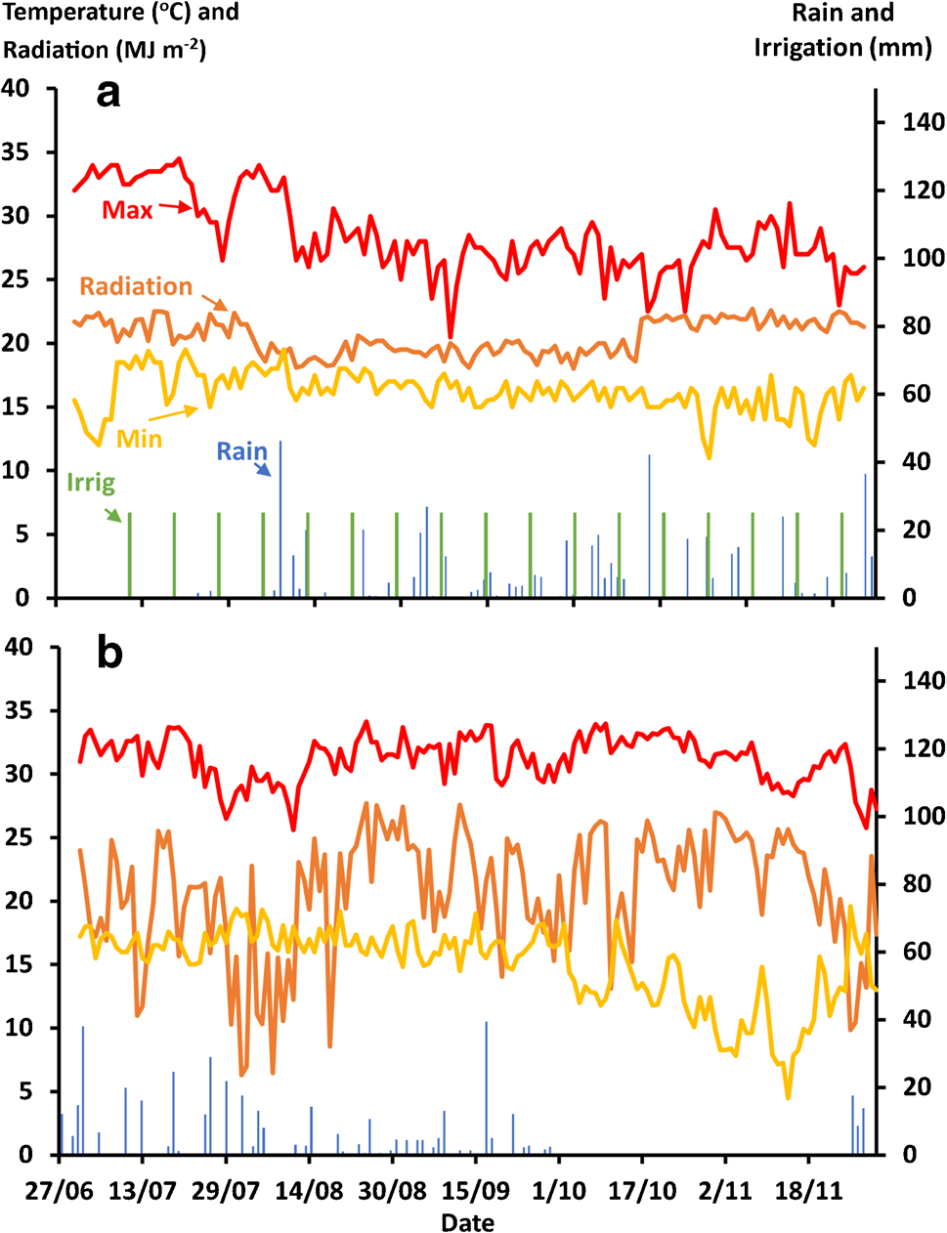
Daily maximum and minimum temperatures, incident solar radiation, and rainfall (plus irrigation at Melkassa) during the field experiments at **a** Melkassa and **b** Miesso.

The daily maximum and minimum temperatures, incident solar radiation, and rainfall (plus irrigation at Melkessa) during the experiment reflected normal seasonal conditions for these sites (Fig. 4).

#### Experiment simulation

**Fig. 5:**
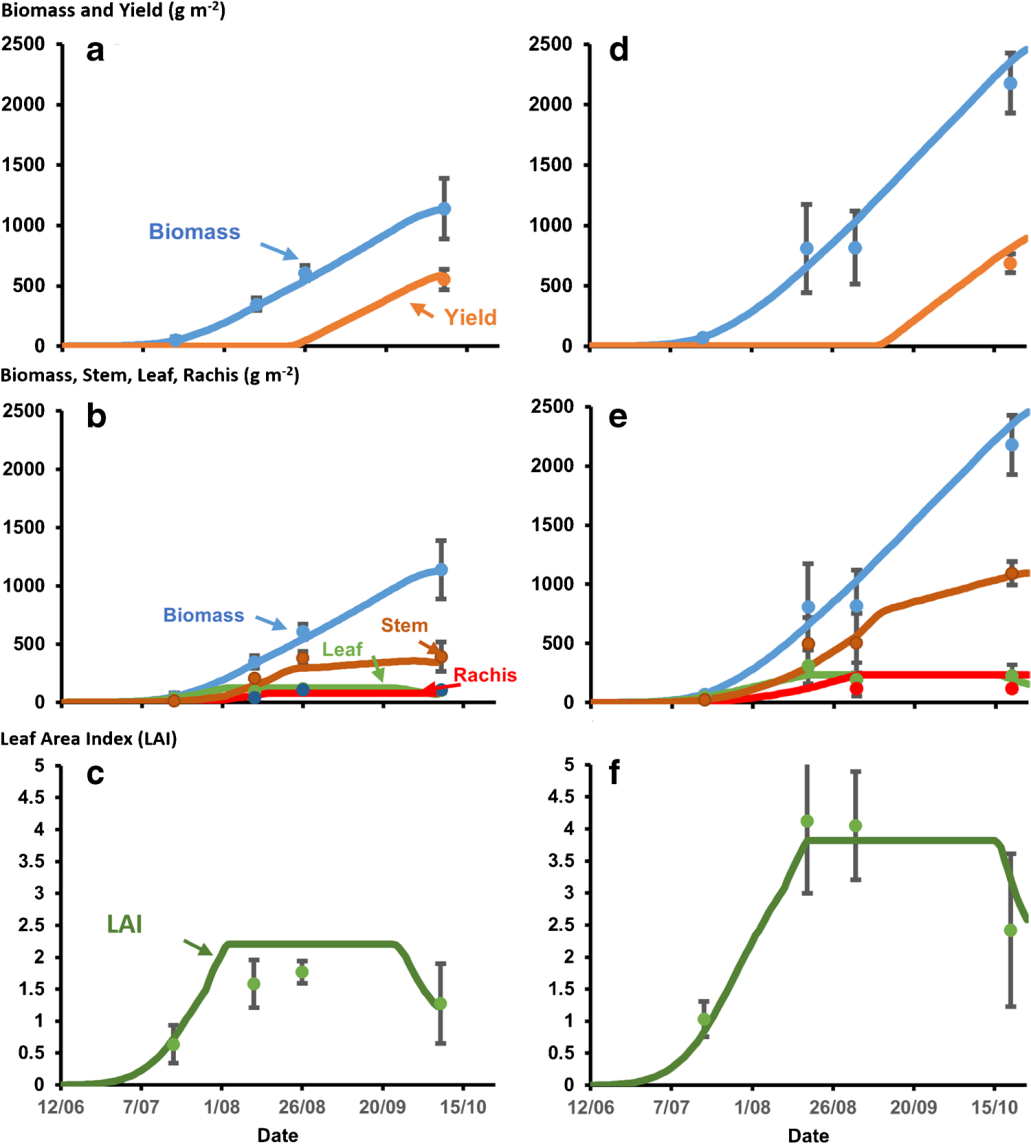
Simulated total biomass and yield (**a**, **d**), biomass components (**b**, **e**), and leaf area index (LAI) (**c**, **f**) for the experiment planted on 10 June 2014 at Melkassa for genotypes Meko (**a**, **b**, **c**) and Jigurti (**d**, **e**, **f**) using the APSIM sorghum model parameterized for those genotypes. Symbols show observed data and vertical bars indicate confidence interval (*p* = 0.10) for associated observations.

**Fig.6:**
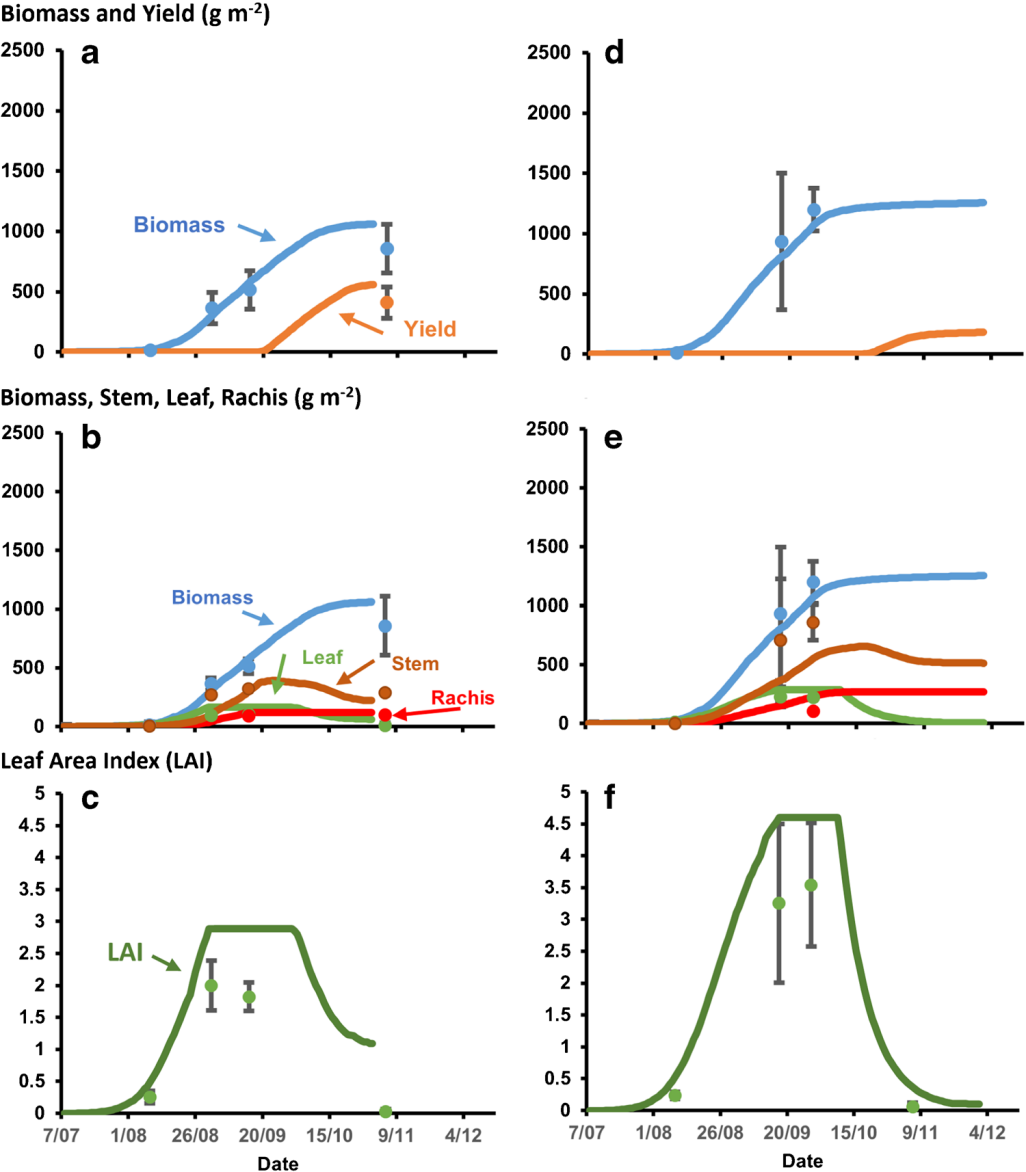
Simulated total biomass and yield (**a**, **d**), biomass components (**b**, **e**), and leaf area index (LAI) (**c**, **f**) for the experiment planted on 08 July 2016 at Miesso for genotypes Meko (**a**, **b**, **c**) and Jigurti (**d**, **e**, **f**) using the APSIM sorghum model parameterized for those genotypes. Symbols show observed data and vertical bars indicate confidence interval (*p* = 0.10) for associated observations. There was no final biomass and yield harvest data for Jigurti due to inability to access the site at that time.

The overall fit of the model was tested by comparing the simulated with observed total biomass, leaf area, and yield for the two genotypes in the two experimental conditions (Fig. 5 and Fig. 6). The APSIM-sorghum model simulated crop growth well for the different plant attributes including biomass, yield, and LAI for Meko and Jigurti under the non-limiting (water and nitrogen) conditions at Melkassa (Fig. 5) with simulated values of total biomass within the error of measurement. The variation between the two genotypes, Meko a shorter stature and early-maturing variety relative to the taller and late Jigurti, was also simulated well in this experiment. The greater LAI for Jigurti was associated with its later maturity and greater leaf number and size (Fig. 3). When combined with its greater RUE, Jigurti was thus predicted to accumulate more total biomass and yield higher. It also had greater stem mass due to its enhanced partitioning associated with its height. However, it also accumulated significant stem mass after anthesis due to the reduced grain set relative to its growth rate generating a grain sink limitation, leading to a lower harvest index than Meko. These findings indicated the adequacy of the coefficients estimated from previous studies on structurally similar genotypes for this potential growth experiment.

Under the dry land conditions in the experiment at Miesso, the APSIM-sorghum model also simulated crop growth well for the different plant attributes (Fig. 6) with simulated values of total biomass within the error of measurement. Both Meko and Jigurti showed enhanced senescence and reduced growth in the latter part of the season compared to the results for the well-watered experiment at Melkessa, due to the depletion of available water. However, this effect was much more pronounced for the late-maturing Jigurti, which had fully senesced by maturity. While the simulation captured these effects in general, they also showed the potential for increased yield of the early-maturing Meko in these circumstances, as the early maturity and lower leaf area enabled some escape from the terminal water limitation. However, Jigurti still produced more total biomass than Meko. The results indicate that the APSIM-sorghum model parameterized for Ethiopian germplasm demonstrated a credible predictive capability for genotype-environment interactions across these diverse situations. While more detailed validation data would be desirable, and the inability to obtain the final biomass and yield harvest for Jigurti at Miesso due to civil unrest was unfortunate, the results provide sufficient confidence in the parameterization and predictive capability of the model to pursue the proposed simulation study.

### Simulation study

#### Production risks associated with early and late plantings

**Fig. 7:**
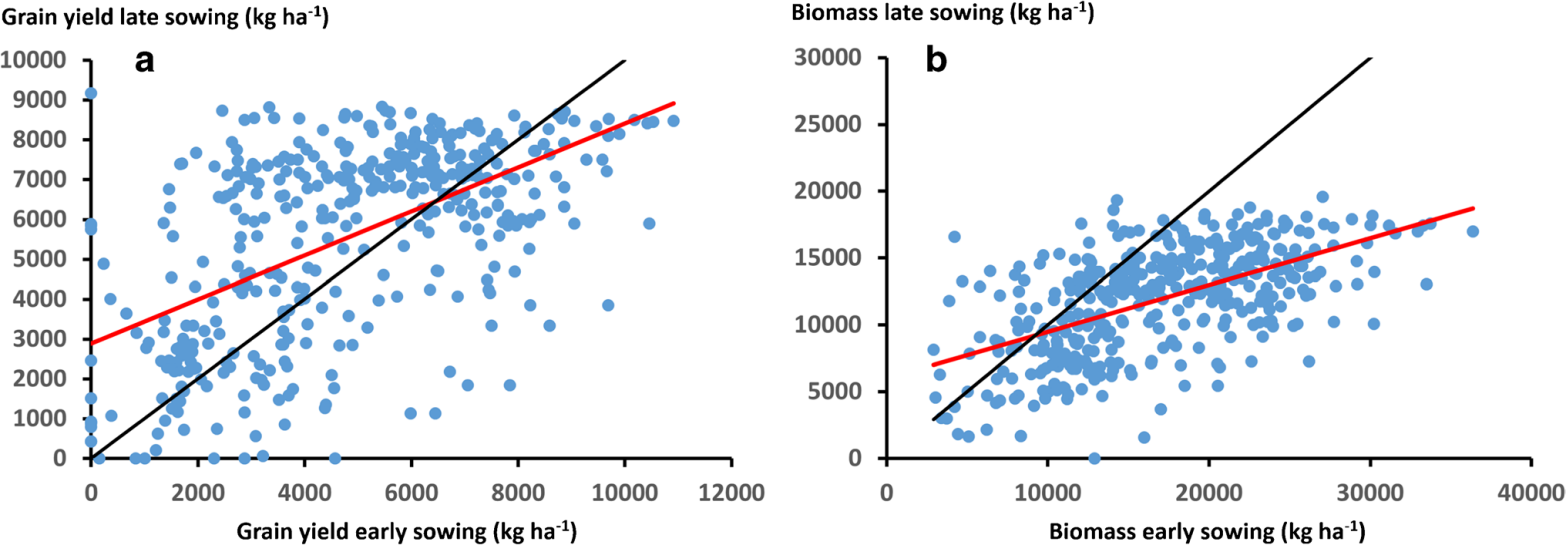
Simulated grain yield (**a**) and total biomass (**b**) for late sowing strategy (late sown early-maturing type) versus early sowing strategy (early sown late-maturing type) for all individual year contrasts in all regions (Oromia, Amhara, Tigray, and Southern) of the Ethiopian dry lowlands. The solid black line is the 1:1 line and the red line the linear regression (**a** *y* = 0.552x + 2890 R^2^ = 0.32; **b** *y* = 0.349x + 6012 R^2^ = 0.33).

The long-term simulation study revealed that when viewed across all regions (Oromia, Amhara, Tigray, and Southern), there was a trade-off between total biomass and grain yield associated with the two production systems of early sowing with a late-maturing tall landrace type, and late sowing with an early-maturing short variety. The late sowing strategy tended to produce greater grain yield except in very good seasons (Fig. 7a), whereas it tended to produce less total biomass except in poor seasons (Fig. 7b). Hence, there was considerably reduced risk associated with the late sowing strategy in poor seasons as it tended to produce both more total biomass and grain yield in those situations.

This overall outcome reflects the interaction of these two production systems with growing season duration and water available to the crops across the production region. The traditional system of early planted late-maturing landrace had a much longer growing season resulting in greater biomass production in most seasons. However, this often resulted in reduced water availability during grain filling and hence, reduced grain yield when compared to the late-planted early-maturing cultivar system. This dominant interaction associated with effects on the water balance would likely also occur with other cultivars with similar maturity characteristics when considered for the entire production region.

**Fig. 8:**
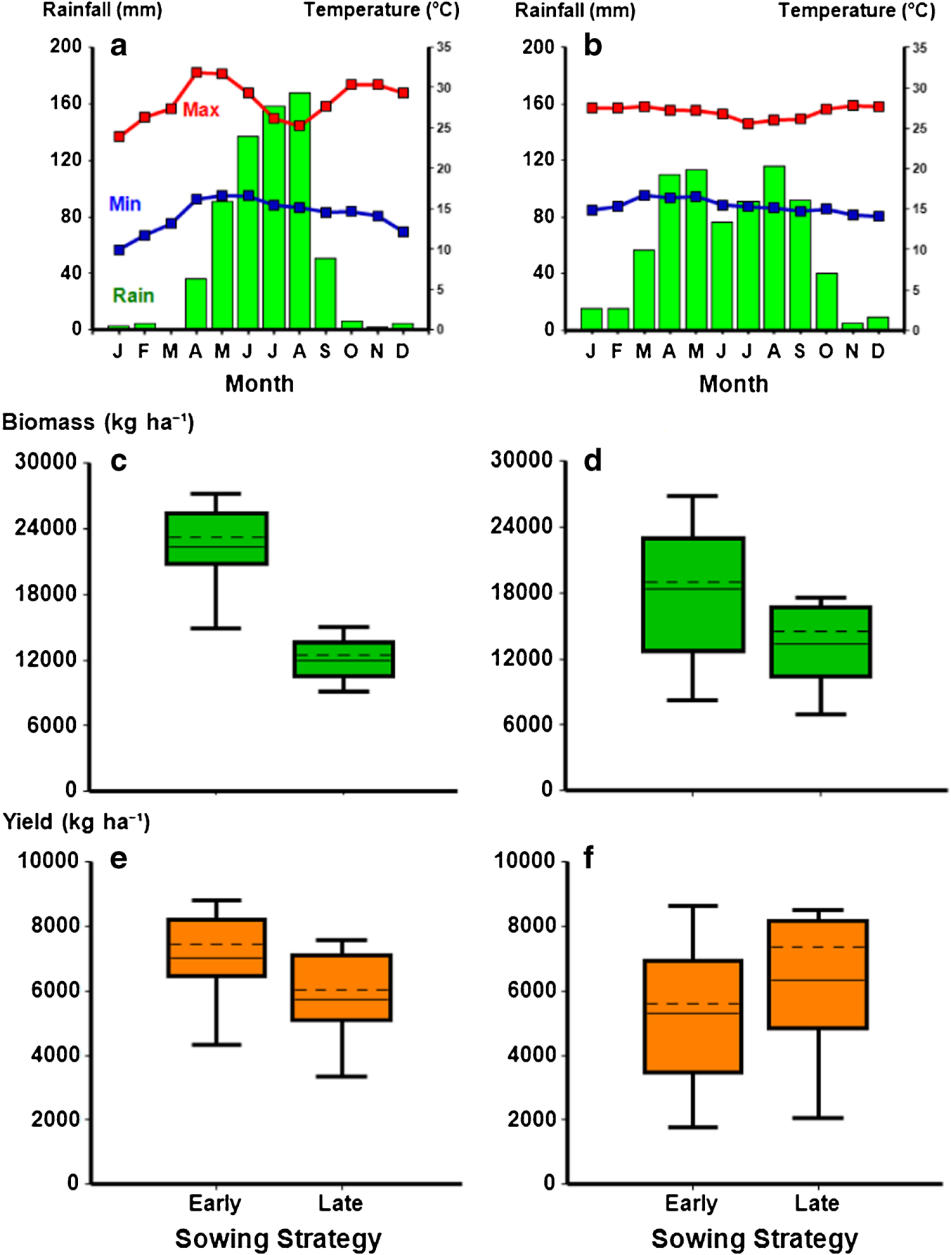
Long-term average monthly rainfall and temperatures (**a**, **b**) and simulated biomass (**c**, **d**) and yield (**e**, **f**) distributions for early and late sowing strategies under the differing rainfall scenarios of the two contrasting regions—Tigray (unimodal—location Shiraro) (**a**, **c**, **e**) and Oromia (bimodal—location Babile) (**b**, **d**, **f**). Boxplots show the median (solid line), mean (dashed line), the 25th and 75th percentile (solid box), and 5th and 95th percentiles (whiskers).

However, considering only the regions of Oromia and Tigray, which show contrasting seasonal patterns of rainfall from unimodal (Tigray) to bimodal (Oromia) (Fig. 8) that are a common features of other locations, the long-term simulation studies showed that there were regional differences in effects on total biomass and yield for the early and late sowing strategies (Fig. 8). Early sowing with the late- maturing variety had better yield and biomass than late sowing with the early-maturing variety in more favorable rainfall environments, such as Tigray. However, the late sowing strategy had better grain yield outcomes in regions with a reduced and bi-modal rainfall pattern (e.g., Oromia) but there was a trade-off with biomass. While the early sowing strategy in the Oromia region gave increased chance of higher biomass than the late sowing strategy, the opposite occurred for grain yield. The long-term simulation studies revealed the production risk trade-offs associated with the late sowing strategy in environments such as Oromia. This type of analysis provides a robust basis for considering the choice among these two systems, or the appropriate mix of the two that might provide some risk reduction.

The simulation analysis quantified the production risk trade-offs associated with early planting with a late- maturing type vs late planting with an early-maturing type in the dry lowland sorghum environments of Ethiopia in a manner not previously done. The sorghum model adapted to Ethiopian conditions could now be used to manipulate/explore the adaptation landscape to better integrate genotype (G), environment (E characterization), management practice (M), and their interactions (G×E×M) as described initially by Cooper and Hammer (1996) to pursue potential sustainable improvements in dry lowland sorghum growing areas of Ethiopia.

Similar long-term simulation studies with relevantly locally adapted versions of the sorghum model in APSIM have quantified the production-risk trade-offs associated with G×E×M interactions for sorghum crop adaptation studies in Australia (Hammer et al. 2014, 2020), the USA (Ojeda et al. 2022), India (Kholova et al. 2013), and Mali (Diancoumba et al. 2022). The basic crop growth and development framework underpinning the APSIM sorghum model thus demonstrates a general robustness for predictions in diverse situations. A key lesson from this study is the ability to effectively utilize this modeling framework for detailed adaptation analysis once some key studies to parameterize key equations for local genotypes have been undertaken and local soil, climate, and agronomic management information has been collated.

## Concluding remarks

The ability to predict biomass and yield across a range of key Ethiopian genotypes and growing/environmental conditions relevant to Ethiopia has generated a capacity not previously available in Ethiopia. While there remains room for improvement by further targeted studies, there is now a credible model for sorghum in Ethiopia that can be used to support more extensive G×E×M studies in crop improvement and adaptation. The quantification of the production risk trade-off presented here for early planting with a late-maturing type versus late planting with an early-maturing type provides information not previously available for farmers and the government about the choice of the system. There was a trade-off between biomass and grain yield across the two systems. In regions such as Oromia with a strong bi-modal rainfall pattern, and where sorghum grain and stover are equally important in the mixed crop-livestock farming system, this type of analysis provides information of direct relevance to the risky decisions faced by farmers. The risk preference of farmers and other influencing factors, such as possible carryover of stored soil water from the previous year and seasonal drought forecasts, will all influence decision-making related to obtaining a target yield and biomass with a specified degree of risk.

The modeling framework and approach used in this study is relevant for sorghum crop adaptation analysis in general. It is particularly important for sorghum production in the dry lowland areas of Ethiopia because the integration of G×E×M has not been explored to date for either broad or specific adaptation. Hence, this integrated systems approach provides relevant quantitative support to the Ethiopian sorghum improvement program to achieve productivity gains.

## Data Availability

The datasets generated during and/or analyzed during the current study are available from the corresponding author on reasonable request.

## Code availability

The APSIM sorghum model is available for download through the APSIM Initiative at www.apsim.info
